# Nerves and Cells

**Published:** 1890-11

**Authors:** 


### NERVES AND CELLS.

A few drops of water from a stagnant pool, when examined under the microscope, reveals the lowest form of life, the study of which is very curious and interesting. These transparent, jelly-like protoplasms are not more than one five-hundredth of an inch in size, and many are less ; yet they move about, eat, and are sensitive to sound or motion. They have no organs of locomotion, yet seem to have the ability of thrusting out feet when they desire to move. They have also power to contract, although possessing no muscles. If one of these minute creatures comes against a spore or germ, it immediately puts out a lip on one side, and then on the other, and soon the germ or spore is on the inside, instead of on the outside, of the protoplasm ; yet it is just as plainly visible as before. This mite of protoplasm has wrapped up in it all the properties of living matter.

The cell life, or protoplasm, of the human body is precisely analagous with that described above, except in size, being only one-fourth as large. These cells are called human amœba, as distinguishing them from other amœba. Each muscular fibre may be considered as a long row of these little, living cells. The liver is made up of just such living cells, each busy making bile ; while some of those of the stomach manufacture gastric juice.

In the cells of the brain and spinal cord, the property of sensibility is chiefly developed, while in the muscles, the contractile property is chiefly developed.

In the brain, these are known as nerve cells, and they send branches down through all parts of the body. Some cells have one, some two, and others a larger number of these thread-like branches or fingers leading from them. A nerve is simply a bundle of these fine threads from the cells, bound all together, just as the wires which go to make up a submarine cable are bound together so as to constitute one insulation. It would take twenty thousand of these little nerve fibers to make a band an inch wide. In the brain there are some three or four trillion of these cells. They are arranged so as to form the outer layer or covering of the brain, and constitute what is called the gray matter ; while the little threads or filaments they send out are deeper in the brain substance, constituting the white matter. These nerve fibers so ramify in all directions that they reach every portion of the body. The point of a pin thrust in the finger would cover a good many of them. In the brain itself these telegraph lines have a great variety of work to do. Some of them have charge of the sense of hearing, and if they become disordered, will cause a person to hear noises which are purely subjective, and not real. The same is true of those which preside over the sense of sight. A story is told of the arrest of one man by another for an assault committed in the dark. The complainant was asked, "How do you know that this is the man who struck you, since the night was pitch dark?" "Well," was the reply, "he struck me in the eye, and made me see stars; and by the light of the stars I saw him!" Of course this was impossible, since the stars he saw were merely subjective visions, not real. Light depends upon the excitement of the cells of the brain, and not necessarily upon the eyes alone.

If a blood clot is formed in the brain, the nerve cells of that part are destroyed, and though the long reaching filaments which lead from that part of the brain to some portion of the body, the arm for instance, are still there, they convey no sensation, and the condition which we call paralysis is induced.

A class of nerve cells of especially vital importance, is that which is capable of receiving impressions sent inward to the brain on the sensory nerves. These cells are not all alike, since through them we experience many different sensations—pain, fatigue, hunger, thirst, heat, cold, etc., besides the special sensations we denominate hearing, seeing, tasting, and smelling, each of which has its special line of nerves connecting the various seats of impression, such as the ear and the eye.

There is another class of cells which has an entirely different property, possessing the ability to send impulses outward. These may be called cells of work. The nerve cells of the brain send down impulses to other cells, from which nerve fibers are distributed to the various muscles, forcing them into activity. The cells of the brain and the spinal cord, as well as the muscles, are constantly storing up energy, to be released as thought or action when the proper stimulus comes. All the work we do, mental or physical, is through this releasing of energy by impulses sent down from the brain. There are similar nerve cells which cause the heart to do its work. One set of cells accelerates its movements when necessary; another restrains it. Bile making and the manufacture of gastric juice are also under control of certain brain cells, as are all other bodily processes.

Then there are other cells which do not produce work, but which regulate work. We go on breathing without taking thought, because there are certain cells which control the breathing automatically. These cells are very sensitive to carbonic acid gas, and when this gas accumulates a little in the body, these cells send down an impulse to the lungs to take in more air, and thus expel the gas which is dangerous to life. This answers for a few seconds, when the cells again recognize that the body is liable to be poisoned unless more air is taken in, and thus send down another impulse to the lungs to act. Sometimes we are nervously excited in some way, perhaps by reading a very interesting book, or listening to an absorbing story, and we forget to breathe as regularly or as deeply as we should, when we are roused up with a deep breath or sigh, which makes up for our remissness. Nature rallies her forces by this deep inspiration.

Other cells of the brain have the power to determine the weight of objects. Still others have the power of balance of movements, being sort of regulators. This regulation of balance is a very remarkable thing. When this is perfect, as in health, the different parts of the body know instinctively their relations, part to part. The finger tips of the two hands can be brought exactly together, though the eyes be closed, the action bringing into play thousands of nerve and muscle cells in harmonious concert. We are able to do this because the two sides of the body are so perfectly made that one part knows where the other part is.

Every part of the muscular system is represented in the gray matter covering the cerebrum. Each little fold or convolution has its corresponding muscle or group of muscles which it controls. Thus is every part of the body, every thought, and power, and action, voluntary or involuntary, governed by some special group of nerve cells set apart for the purpose.—*Good Health.*

---

### HERBA-VITA.

Having had the most positive assurances of the harmlessness and efficacy of the above remedy for the "ills that flesh is heir to," we have regarded its action in a number of instances and are glad to be able to endorse its claims as a blood purifier and rectifier of clogged and disordered systems proceeding from whatever cause, but particularly from a too free use of poisonous drugs. It is a veritable assistant of nature in restoring good health.

